# Anaplastic Sarcoma of the Kidney in a Child with *DICER1* Syndrome: A Case Report

**DOI:** 10.70352/scrj.cr.25-0010

**Published:** 2025-08-14

**Authors:** Eri Nagasaki-Maeoka, Katsuyoshi Shimozawa, Masaru Ueno, Kanako Saiki, Hiroshi Yagasaki, Haruna Nishimaki-Watanabe, Ryusuke Tsujimura, Yoshiko Nakano, Motohiro Kato, Tsugumichi Koshinaga, Shuichiro Uehara

**Affiliations:** 1 Department of Pediatric Surgery, Nihon University School of Medicine, Tokyo, Japan; 2 Department of Pediatric Surgery, Jichi Medical University, Saitama Medical Center, Saitama, Saitama, Japan; 3 Department of Pediatrics, Nihon University School of Medicine, Tokyo, Japan; 4 Division of Oncologic Pathology, Department of Pathology and Microbiology, Nihon University School of Medicine, Tokyo, Japan; 5 Division of Human Pathology, Department of Pathology and Microbiology, Nihon University School of Medicine, Tokyo, Japan; 6 Department of Pediatrics, the University of Tokyo, Tokyo, Japan

**Keywords:** anaplastic sarcoma of the kidney, children, rare renal tumor, *DICER1*

## Abstract

**INTRODUCTION:**

Anaplastic sarcoma of the kidney (ASK) is a rare renal tumor, with fewer than 50 cases reported in the literature since 2007. ASK is pathologically characterized by the presence of cystic and solid areas consisting of spindle cells showing marked anaplasia. Recent studies have reported that the vast majority of patients with ASK have *DICER1* variants, and that these tumors are part of the *DICER1* syndrome, a hereditary cancer predisposition disorder. Herein, we report a pediatric case of this rare tumor, including pathological findings, *DICER1* gene analysis of the tumor and peripheral blood samples, and the disease course.

**CASE PRESENTATION:**

A previously healthy 2-year-old girl presented with gross hematuria and a mass in her right abdomen. She had a family history of tumor; her eldest maternal aunt had developed rhabdomyosarcoma, another maternal aunt had follicular thyroid cancer, and her maternal grandmother had a benign thyroid tumor. Imaging revealed a 10-cm tumor with conspicuous internal cystic structures in the right kidney. The patient underwent right nephrectomy, removing a tumor measuring 12.5 × 9 × 8 cm that contained cystic and solid parts. The tumor was composed of spindle-shaped cells with anaplastic changes. Finally, the diagnosis of ASK was established. The treatment regimen, in accordance with the therapy for clear cell sarcoma of the kidney or diffuse anaplasia type Wilms tumor, was administered. Based on the diagnosis of ASK and the family history of *DICER1*-associated tumors, *DICER1* syndrome was suspected. Sequencing of the hotspot region (i.e., RNase IIIb domain) using tumor specimen and coding regions using blood sample in the *DICER1* revealed somatic c.5125G>A (p.D1709N) and germline c.4458dupA (p.S1487Ifs*5). Two years have passed since the end of treatment, and the patient is alive and healthy without evidence of tumor recurrence.

**CONCLUSIONS:**

Treatment with intensive chemotherapy (vincristine, doxorubicin, etoposide, and cyclophosphamide) and abdominal irradiation was effective as 2-year event-free survival was achieved. Since *DICER1* syndrome causes a variety of rare cancers, particularly in infants and young adults, all surgeons and pediatric oncologists should be aware of the possibility of *DICER1* syndrome.

## Abbreviations

ASK
: anaplastic sarcoma of the kidney
CCSK
: clear cell sarcoma of the kidney
CN
: cystic nephroma
EFS
: event-free survival
JCCG
: Japan Children's Cancer Group
JWiTS
: Japan Wilms Tumor Study
NSS
: nephron-sparing surgery
NWTS
: National Wilms Tumor Study
PPB
: pleuropulmonary blastoma
VDC-IE
: alternating chemotherapy consisting of vincristine, doxorubicin, and cyclophosphamide, along with ifosfamide and etoposide
WT
: Wilms tumor

## INTRODUCTION

ASK is a rare and relatively newly recognized renal tumor in pediatric and younger patients. To date, fewer than 50 cases have been reported in the literature since 2007 following the first description by Vujanić et al. ^1–17)^. Due to its rarity, no standard treatment exists, and little is known about its prognosis. *DICER1* syndrome, caused by pathogenic germline variants of *DICER1*, predisposes individuals to the development of various tumors.^18)^ Recent studies have suggested that ASK belongs to the category of *DICER1*-associated tumors.^10,15,18,19)^

Here, we report a case of this rare tumor in a 2-year-old girl with a family history of cancer that was found to have *DICER1* variants.

## CASE PRESENTATION

**Fig. 1:**
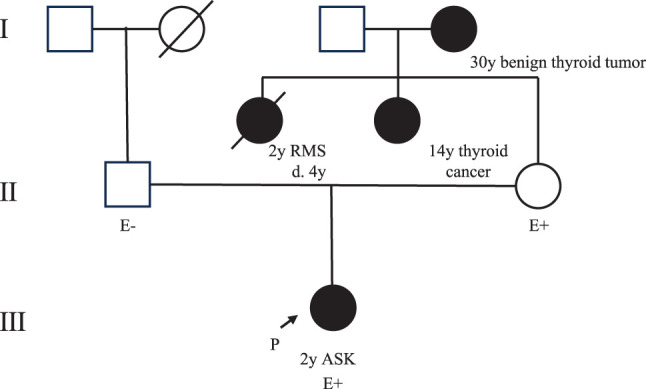
Pedigree chart. The patient’s eldest maternal aunt developed RMS at 2 years of age and died of her illness at the age of 4 years. Another maternal aunt had follicular thyroid cancer at 14 years of age, and the patient’s maternal grandmother had a benign thyroid tumor at age 30 years. The 2-year-old patient was diagnosed with ASK. As cascade testing, we performed genetic analyses using samples from the patient’s parents and found that her mother had the same germline variant. ASK, anaplastic sarcoma of the kidney; E, evaluation; P, proband; RMS, rhabdomyosarcoma

**Fig. 2:**
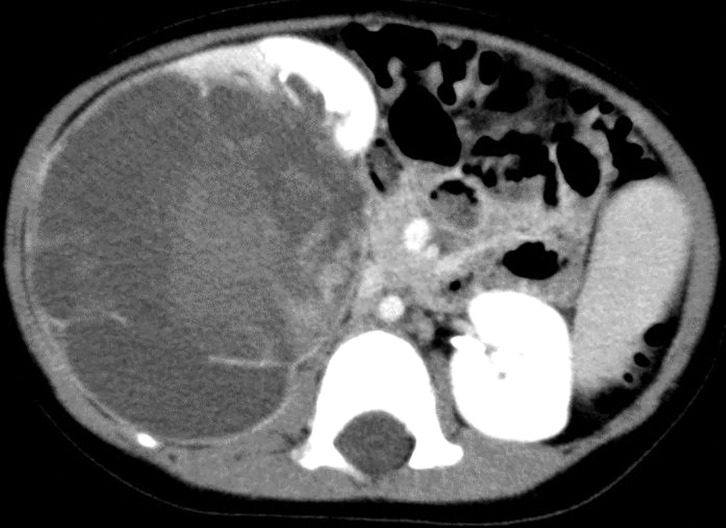
Contrast enhanced computed tomography of the abdomen and pelvis. A 10-cm multicystic tumor with a solid component is detected in the right kidney.

A previously healthy 2-year-old girl presented with gross hematuria and a mass in her right abdomen and was referred to our hospital. Regarding her family history, her eldest maternal aunt developed rhabdomyosarcoma at 2 years of age and died of her illness at the age of 4 years. Furthermore, another maternal aunt had follicular thyroid cancer at 14 years old, and her maternal grandmother had a benign thyroid tumor at 30 years old (**Fig. 1**). Physical examination revealed that her abdomen was distended, and an elastic-hard mass, 10 cm in diameter, was palpable in her right abdomen. Laboratory findings revealed no abnormalities, including tumor marker levels, except for high levels of lactic dehydrogenase (527 U/L, reference level 124–222), neuron-specific enolase (39.5 ng/mL, reference level ≤16.3), and urine occult blood (3+). Abdominopelvic contrast-enhanced CT revealed a 10-cm tumor in the right kidney. The tumor was multicystic with a solid component (**Fig. 2**). Abdominal magnetic resonance imaging indicated that the fluid component of the cyst was hyperintense on both T1- and T2-weighted images. No distant metastases were observed. Based on these findings, the possible differential diagnoses included clear CCSK, malignant rhabdoid tumor of the kidney, cystic WT, multilocular CN, and congenital mesoblastic nephroma.

**Fig. 3:**
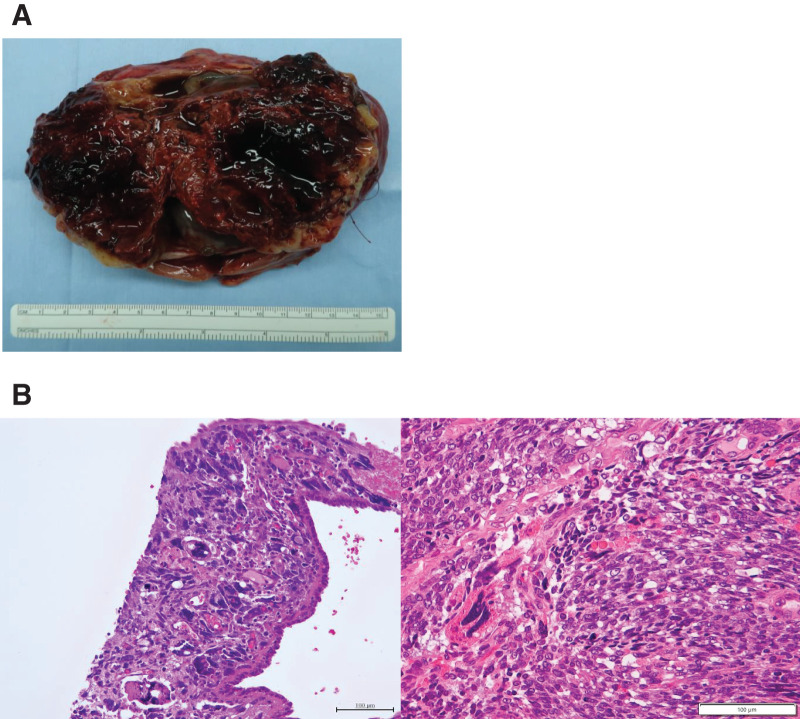
Gross and microscopic findings of the tumor. (**A**) Macroscopic view of the tumor. The cut surface of the tumor contained cystic and solid components, some of which appear necrotic. (**B**) Hematoxylin and eosin staining. The tumor consists of spindle-shaped cells with some anaplastic changes.

Right nephrectomy was performed and the tumor was completely resected without rupture. The tumor removed from the right kidney measured 12.5 × 9 × 8 cm and weighed 472 g (**Fig. 3A**). The cut surface of the tumor contained cystic and solid parts, some of which were considered necrotic. The histologic examination revealed that the tumor was composed of spindle-shaped cells with some anaplastic changes (**Fig. 3B**). Immunostaining examinations revealed that the tumor was weakly positive for BCOR; negative for WT-1, myogenin, and SMA; and positive for INI-1 in the nucleus.

The patient was tentatively diagnosed with CCSK (NWTS stage II). Based on this diagnosis, chemotherapy and radiotherapy were initiated in accordance with the JWiTS protocol regimen (vincristine, doxorubicin, etoposide, cyclophosphamide, and 10.8 Gy abdominal irradiation).^20)^ Nine weeks after the diagnosis, the results of the central review of the JCCG indicated that BCOR did not exhibit a clear positive nucleus image upon immunostaining, with a solid/cyst component and fascicular pattern observed, leading to a diagnosis of ASK. Chemotherapy continued unchanged and was completed without severe adverse events. The total doses of chemotherapeutic agents were as follows; vincristine 0.718 mg/kg, doxorubicin 7.5 mg/kg, etoposide 66 mg/kg, and cyclophosphamide 470.4 mg/kg. The treatment duration was 180 days.

Based on the family history of cancer, *DICER1* syndrome was suspected. Genetic analyses revealed variants in the *DICER1* gene in the tumor tissue (c.5125G>A) and peripheral blood (c.4458dupA) (**Table 1**). Two years have elapsed since the end of treatment, and neither recurrence of ASK nor occurrence of other tumors has been observed.

**Table 1:** Results of *DICER1* gene analyses

Sample	Detected variant
Tumor	c.5125G>A, p.D1709N
Peripheral blood	c.4458dupA, p.S1487Ifs*5

## DISCUSSION

Pediatric renal tumors account for 7% of childhood cancers.^21)^ The most common childhood renal tumor, accounting for approximately 90% of cases, is WT, followed by CCSK, renal cell carcinoma, rhabdoid tumor of the kidney, and other rare tumors.^22)^ Therapy and prognosis differ among these subtypes; therefore, an accurate diagnosis is important. Because no molecular biomarkers or specific radiological findings exist for childhood renal tumors, histological evaluation is needed to distinguish these subtypes. ASK is a rare renal tumor, with fewer than 50 cases reported in the literature.^1–17)^ A summary of ASK cases is shown in **Table 2**. It is characterized by the presence of cystic and solid components consisting of spindle cells, indicating marked anaplasia.^1)^ Anaplastic WT is the most important tumor in the differential diagnosis.^1,4,12)^ WT usually appears as a large, heterogeneous intrarenal mass on abdominal CT, demonstrating a lower degree of enhancement compared with the adjacent normal renal parenchyma.^23,24)^ Necrosis and hemorrhage are commonly observed in the intratumoral portion, and calcification is seen in 9% of WT.^25,26)^ On the other hand, ASK appears as a multi-loculated cystic tumor in some previously reported cases.^7,12)^ It is reported that ASK is highly suggested by discordant image findings on sonography or CT scan; namely, the presence of predominant hyperechoic solid components on sonography, but a mainly low-density cystic appearance on CT scan.^12)^ Based on the characteristic imaging study and family history of this case, ASK and CN would have been considered the most likely differential diagnoses prior to surgery; however, it would have been difficult to make a definitive diagnosis without conducting a histopathological examination. In the present case, anaplastic WT was ruled out based on the negative results of immunohistochemical staining for WT1. The characteristic finding of CCSK is Orphan Annie-eye nuclei^27)^; therefore, in the absence of such a finding, being cautious when making a diagnosis is recommended. Additionally, weakly positive BCOR immunostaining did not provide a basis for the diagnosis of CCSK. ASK should have been listed as a differential diagnosis from a clinical point of view and carefully judged for whether it was consistent with the histological findings.

**Table 2:** Summary of ASK cases including the present case

Total cases	48
Age in years, mean	11.4
Age in years, median	8.4
Sex	M:16, F:32
Stage	
I	14 (29%)
II	13 (27%)
III	10 (21%)
IV	5 (10%)
No data	6 (13%)
Chemotherapy	
Yes	39 (81%)
No	8 (17%)
No data	1 (2%)
Radiotherapy	
Yes	15 (31%)
No	23 (48%)
No data	10 (21%)
Relapse	
Yes	10 (21%)
No	29 (60%)
No data	9 (19%)
Status	
NED	29 (60%) (observation time: 9 months–14 years)
AWD	4 (8%)
DOD	6 (13%)
No data	9 (19%)

ASK, anaplastic sarcoma of the kidney; AWD, alive with disease; DOD, died of disease; F, female; M, male; NED, no evidence of disease

While no standard therapy for ASK has been established, nephrectomy combined with adjuvant chemo-radiotherapy is the treatment of choice for most patients. Recently, many patients have received sarcoma-oriented regimens such as an alternating chemotherapy consisting of vincristine, doxorubicin, and cyclophosphamide, along with ifosfamide and etoposide (VDC-IE).^14,17)^ The clinical outcome of patients with ASK has been variable, but correlates with tumor staging.^11,15,17)^ Two-year EFS for stage I–II ASK was marginally better than that for stage III–IV (81.8% vs. 46.6%, *P* = 0.07). Two-year overall survivals for stage I–II and stage III–IV patients were 88.9% and 70.0%, respectively (*P* = 0.20).^17)^ Radiotherapy for ASK is considered acceptable as in previously published case reports, radiotherapy was performed on 31% of ASK patients, with most showing no evidence of disease at follow-up (**Table 2**). In addition, the effectiveness of radiotherapy for tumors associated with *DICER1* syndrome other than ASK has been reported.^28–30)^ Although no standard treatment for ASK has been established yet, the intensive regimen (vincristine, doxorubicin, etoposide, and cyclophosphamide) with radiotherapy was effective in our case as 2-year EFS was achieved.

*DICER1* syndrome is an autosomal dominant hereditary cancer predisposition disorder caused by germline pathogenic variants of *DICER1*.^31–33)^ Tumors that present with *DICER1* syndrome typically have a germline nonsense or frameshift pathogenic gene variant in 1 allele and a somatic hotspot variant in RNase IIIb domain in the other, the latter of which is thought to lead to tumorigenesis.^19,31,34,35)^ In the present case, c.5125G>A (p.D1709N) was detected in tumor and c.4458dupA (p.S1487Ifs*5) was detected in germline. As cascade testing, we performed genetic analyses using samples from the patient’s parents and found that her mother had the same germline variant. These results indicate that the tumor tissue had a germline variant p.S1487Ifs*5 in 1 allele and then acquired the missense variant p.D1709N, resulting in the onset of ASK as a manifestation of *DICER1* syndrome.

Tumors that can occur in *DICER1* syndrome include pleuropulmonary blastoma, thyroid tumors, CN, rhabdomyosarcoma, and ovarian sex cord-stromal tumors.^18,19)^ Prior information that a tumor associated with *DICER1* syndrome is suspected may affect the surgical strategy and histological diagnosis in some types of cancer. For example, if type I PPB is suspected, then a total resection should be planned to prevent dissemination, and the entire specimen should be carefully examined, as undifferentiated cells may be present in some areas of the thin tumor wall.^36,37)^ In our case, prior information that a tumor associated with *DICER1* syndrome was suspected would not have led to any change in the surgical procedure, because the standard surgical procedure for pediatric unilateral kidney tumors is nephrectomy on the affected side to completely remove the tumor without causing it to rupture. However, if a tumor associated with *DICER1* syndrome had been suspected prior to surgery, then a diagnosis of anaplastic sarcoma of the kidney might have been made from the beginning. In addition, since rare diseases such as PPB are often difficult to diagnose based on the pathological findings alone, the detection of pathogenic variants via an analysis of tumor hotspots can be a useful finding leading to a definitive diagnosis.^38)^

As *DICER1* syndrome is a cancer predisposition syndrome, we should consider surgical treatment with the aim of preserving organs as much as possible. In Japan, treatment of childhood renal tumors is often carried out in accordance with the JWiTS protocol; generally, NSS is not performed in cases involving a unilateral tumor. However, NSS is now acceptable for nonsyndromic unilateral WT under certain conditions, specified in the international new protocol for diagnosis and treatment of childhood renal tumors. These include a small tumor volume (<300 mL) and the expectation of substantial remnant kidney function in patients with tumors <300 mL who have never had lymph node involvement.^39)^ It would also be acceptable to perform NSS in ASK according to this WT protocol.

Patients with *DICER1* syndrome have been reported to develop multiple tumors including thyroid and ovarian tumors in their lifetime.^31)^ Schultz et al. have recommended surveillance strategies for individuals with *DICER1* pathogenic variants.^35)^ With reference to their surveillance strategies, in our case, follow-up examinations are conducted via regular check-ups as follows: 1) Chest X-rays for lung check-ups every 4–6 months until the age of 8 years, then annually from age 8 to 12 years; 2) Thyroid ultrasounds for thyroid check-ups once before the age of 8 years, then every 2–3 years; 3) Abdominal and pelvic ultrasounds for reproductive organ check-ups every 6 months to 1 year from age of 8 to 40 years; and 4) Abdominal ultrasounds for kidney check-ups every 6 months until age of 8 years, then annually until age of 12 years. Schultz et al. suggest detecting signs and symptoms and imaging surveillance by system or organ.^35)^ Therefore, these surveillance strategies are useful for other cancers in various organs.

## CONCLUSIONS

We encountered the case of a child with *DICER1* variants who developed ASK. Although no standard treatment for ASK has been established yet, VDC-IE type regimen was effective, as 2-year EFS was achieved. Prior information that a tumor associated with *DICER1* syndrome is suspected may influence the surgical strategy and histological diagnosis in childhood cancer. Since *DICER1* syndrome causes a variety of rare cancers in young age groups (infants through to young adults), all surgeons and pediatric oncologists should be aware of the possibility of encountering *DICER1* syndrome.

## ACKNOWLEDGMENTS

We would like to thank Ms. Masayo Matsumura for her excellent technical assistance. We also thank Editage (www.editage.jp) for English language editing.

## DECLARATIONS

### Funding

This research was supported by Japan Agency for Medical Research and Development under Grant No. JP24ck0106876; and Gold Ribbon Network.

### Authors’ contributions

ENM drafted the manuscript.

ENM, SU, KShimozawa, MU, KSaiki, HY, and TK analyzed and interpreted the patient data regarding ASK and *DICER1* syndrome.

HNW and RT performed histological diagnoses.

YN and MK performed genetic analysis.

SU critically revised the manuscript for intellectual content.

All the authors have read and approved the final version of the manuscript.

### Availability of data and materials

The datasets used and/or analyzed in the current study are available from the corresponding author upon reasonable request.

### Ethics approval and consent to participate

This study was approved by the Ethics Committee of Nihon University School of Medicine, Tokyo, Japan (Approval No.: MF-2112-0010). Genetic analysis was performed with consent to enroll in the pilot study of pediatric cancer predisposition syndrome (pCPS21). Written informed consent was obtained from the patient’s parents for the participation in this study.

### Consent for publication

Written informed consent was obtained from the patient’s parents for the publication of this case report.

### Competing interests

The authors declare that they have no competing interests.
